# GABA Transporter-1 Deficiency Confers Schizophrenia-Like Behavioral Phenotypes

**DOI:** 10.1371/journal.pone.0069883

**Published:** 2013-07-29

**Authors:** Zhe Yu, Qi Fang, Xian Xiao, Yi-Zhi Wang, You-Qing Cai, Hui Cao, Gang Hu, Zhong Chen, Jian Fei, Neng Gong, Tian-Le Xu

**Affiliations:** 1 Institute of Neuroscience and State Key Laboratory of Neuroscience, Chinese Academy of Sciences, Shanghai, China; 2 Departments of Anatomy and Embryology, Biochemistry and Molecular Cell Biology, Shanghai Key Laboratory for Tumor Microenvironment and Inflammation, Institute of Medical Sciences, Shanghai Jiao Tong University School of Medicine, Shanghai, China; 3 College of Pharmaceutical Sciences, Zhejiang University, Hangzhou, China; 4 School of Life Science and Technology, Tongji University, Shanghai, China; 5 Department of Anatomy, Histology & Pharmacology, Nanjing Medical University, Nanjing, China; University of Chicago, United States of America

## Abstract

The mechanism underlying the pathogenesis of schizophrenia remains poorly understood. The hyper-dopamine and hypo-NMDA receptor hypotheses have been the most enduring ideas. Recently, emerging evidence implicates alterations of the major inhibitory system, GABAergic neurotransmission in the schizophrenic patients. However, the pathophysiological role of GABAergic system in schizophrenia still remains dubious. In this study, we took advantage of GABA transporter 1 (GAT1) knockout (KO) mouse, a unique animal model with elevated ambient GABA, to study the schizophrenia-related behavioral abnormalities. We found that GAT1 KO mice displayed multiple behavioral abnormalities related to schizophrenic positive, negative and cognitive symptoms. Moreover, GAT1 deficiency did not change the striatal dopamine levels, but significantly enhanced the tonic GABA currents in prefrontal cortex. The GABA_A_ receptor antagonist picrotoxin could effectively ameliorate several behavioral defects of GAT1 KO mice. These results identified a novel function of GAT1, and indicated that the elevated ambient GABA contributed critically to the pathogenesis of schizophrenia. Furthermore, several commonly used antipsychotic drugs were effective in treating the locomotor hyperactivity in GAT1 KO mice, suggesting the utility of GAT1 KO mice as an alternative animal model for studying schizophrenia pathogenesis and developing new antipsychotic drugs.

## Introduction

Schizophrenia is a highly debilitating mental disorder that affects approximately 1% of the world's population, which pathogenesis mechanisms remain unclear. Traditionally, the hyper-dopamine hypothesis [1] and the hypofunction of NMDA receptor (NMDAR) [2] are considered as two of the most enduring ideas in schizophrenia. Recently, more and more evidence implicates GABAergic neurotransmission plays an important role in schizophrenia. Postmortem studies report reduced mRNA level and expression of the GABA synthesizing enzyme, 67 kDa isoform of glutamic acid decarboxylase (GAD67), and GAT1, as well as an apparent upregulation of postsynaptic GABA_A_ receptors (GABA_A_Rs) in the prefrontal cortex of human subjects with schizophrenia [3], suggesting a mechanism for abnormal GABAergic neurotransmission in schizophrenia. However, these studies mainly focus on the morphological examinations in individuals with schizophrenia, but the functional study falls far behind.

The GAT1 is primarily responsible for the removal of GABA from the synaptic cleft and termination of GABAergic neurotransmission. It belongs to high-affinity, sodium- and chloride-dependent GABA transporters, and is predominantly abundant in GABAergic neurons [4], [5]. The GAT1 activity plays a crucial role in controlling ambient GABA concentration, modulating both phasic and tonic GABA inhibition [6], [7], [8], [9], [10]. In individuals with schizophrenia, the downregulation of GAT1 was observed in several brain areas, including prefrontal cortex [11], limbic system [12] and cerebellum [13], suggesting reduced GABA reuptake in schizophrenia. However, due to the concurrent downregulation of GAD67, the overall change of GABA level in schizophrenia is quite controversial. Indeed, the literature on GABA measurements in schizophrenia is more discrepant, with reports of either normal [14], [15], reduced [16], [17], [18] or elevated GABA levels [19], [20]. Although it was supposed in several studies that the GAT1 downregulation may be a compensatory mechanism to the reduction of GABA synthesis [3], [21], the functional significance of GAT1 downregulation remains unknown.

In this study, we found that GAT1 KO mice displayed multiple schizophrenia-like behaviors, suggesting that GAT1 downregulation may be a pathogenic mechanism, but not a simple compensatory change. Moreover, the striatal dopamine levels were unchanged in GAT1 KO mice, but the tonic GABA currents in prefrontal cortex were significantly increased. The GABA_A_ receptor antagonist picrotoxin could effectively ameliorate several behavioral defects of GAT1 KO mice. These results underscore the significance of elevated ambient GABA in the pathogenesis and treatment of schizophrenia. Moreover, we found that several commonly used antipsychotic drugs were effective in treating the locomotor hyperactivity in GAT1 KO mice, suggesting the utility of GAT1 KO mice as an alternative animal model for studying schizophrenia pathogenesis and testing new antipsychotic drugs.

## Materials and Methods

### Animals

The care and use of animals in these experiments followed the guidelines of, and the protocols were approved by, the Institutional Animals Care and Use Committee of the Institute of Neuroscience, Shanghai Institutes for Biological Sciences, Chinese Academy of Sciences. The mGAT1 KO strain was used in this study. The details of the targeting construct, homologous recombination, and genotyping were described previously [22]. GAT1 KO mice were backcrossed for 9 generations to C57BL/6J mice. The heterozygotes (HET) were intercrossed to generate homozygous, heterozygous, and wild-type (WT) littermate mice. They were weaned at the fourth postnatal week and their genotypes were analyzed by preparing tail DNAs and PCR assay [22]. Mice were kept at a 12 h light/dark cycle, and the behavioral experiments were always done during the light phase of the cycle. Mice had access to food and water *ad libitum* except during tests. In all experiments, the investigators were blind to the genotype of mice. The experiments were performed on the mice in a randomized order.

In the following experiments, mice were only used for one time: the drug treated experiments, prepulse inhibition, latent inhibition, Morris water maze, *in vivo* microdialysis, *in vitro* electrophysiology and Western blot tests. For observation-based behavior tests, i.e. open field tests without drug administration, novel object recognition, nesting behavior, social interaction, and Y-maze spontaneous alternation, mice were used repeatedly with a certain time intervals.

### Behavioral tests

Adult WT and GAT1 KO male mice (3–5 months old littermates) were used in most of the behavioral tests and 4–5 weeks old mice (young mice) were used to investigate the emergence time of the schizophrenia-like phenotypes. For the drug treatment in behavioral tests, mice were injected intraperitoneally (i.p.) in a volume of 10 ml/kg body weight.

#### Open field

Mice were placed in the center of a square Plexiglas open field apparatus (40×40×35 cm) and allowed to freely explore for 30 min. The same procedure was repeated once daily for 4 consecutive days. Total distance traveled was quantified using the Ethovision videotracking system (Noldus Information Technology, Netherlands). To test the stimulant effects of NMDAR antagonist MK801 and phencyclidine (PCP), mice were injected i.p. with drug after 30-min acclimation in the open field, and then immediately tested for another 30 min. To test the effects of antipsychotic drugs and picrotoxin (PTX), mice were not acclimated to the open field but directly injected i.p. with drug 30 min before the test.

#### Response to novelty and object recognition

Mice were individually habituated to an open field box (50×50×35 cm) before test. In the first day, two different objects (A and B) were used as acute novelty and placed into two distinct quadrants of the open field and each mouse was allowed to explore for 5 min. The object recognition was tested 24 h later. The mice were placed back into the same box, in which one of the familiar objects (A) was replaced by a novel object (C), and allowed to explore freely for 5 min. The time spent in the proximity of the object (the quadrant with object) was measured using Ethovision.

#### Nesting behavior

Ten pieces of the nesting material, made of cotton fiber, was introduced into an empty cage. Then, a mouse was placed in the cage with food and water for an overnight period. Pictures of the nests were taken by a digital camera and exported into a computer. The number of cotton particles was counted for each cage.

#### Social interaction

Social interaction was measured by placing the mouse in a clean empty cage for 60-min acclimation and then introducing an unfamiliar young male C57BL/6J mouse (3-week old) for 10 min. Sniffing, nosing, grooming, and crawling over each other are considered social interest behavior. The duration of social interest behaviors carried out by the resident mouse was recorded by an experienced observer who remained blind to the animal's genotype.

#### Prepulse inhibition (PPI)

A startle reflex measurement system was used (MED Associates, USA) in the PPI test. During the test, the animal was confined to the holder. Background noise was set at 65 dB. Five types of trials were used. Pulse alone trials (P) consisted of a single white noise burst (120 dB, 40 ms). The prepulse+pulse trials (PP70P, PP75P, PP80P) consisting of a prepulse of noise (20 ms at 70, 75, or 80 dB, respectively) followed by a startling pulse (120 dB, 40 ms) 100 ms after prepulse onset. No-stimulus (NS) trials consisted of background noise only. Sessions were structured as follows: (1) 5-min acclimation at background noise level; (2) five P trials; (3) 10 blocks each containing all five trials (P, PP70P, PP75P, PP80P, NS) in pseudorandom order; and (4) five P trials. Inter-trial intervals were distributed between 12 and 30 s. The force intensity for each trial was recorded as the startle level. Startle magnitude in this formula was calculated as the average response to all of the pulse alone trials, excluding the first and last blocks of five pulse alone trials. The percentage PPI induced by each prepulse intensity was calculated as [1-(startle amplitude on prepulse trial)/(startle amplitude on pulse alone)]×100%.

#### Latent inhibition (LI)

Latent inhibition was measured by cued fear conditioning test. The fear conditioning test was performed by a Near Infrared (NIR) Video Fear Conditioning System (MED Associates, USA). During training session, each mouse was placed into the conditioning chamber for 3 min. The mice were divided into two groups: pre-exposed (P) group and non pre-exposed (NP) group. The P group received 40 white noise tones (80 dB, 5 s, 30 s intervals), whereas the NP group received no stimulus during an equivalent period. Immediately after the tone pre-exposure or the exposure to the chamber, tone-shock pairs consisting of a 5 s tone co-terminating with a 2 s foot shock at 0.70 mA were delivered to both groups with a 30 s inter-stimulus interval. During the retention test 24 h later, the mice were put in a white Plexiglas chamber different from the conditioning chamber, and after 180 s, a 180 s tone was delivered to measure cued freezing.

#### Morris Water maze

The Morris water maze consisted of a circular pool (100 cm diameter, 50 cm deep) filled with water at 24–26°C to a depth of 20 cm. The water surface was covered with floating white resin beads. Yellow curtains were drawn around the pool (50 cm from the pool periphery) and contained distinctive visual marks that served as distal cues. Before training, a 60 s free swim trial without the platform was run. In this test, each mouse was given four consecutive 60-sec training trials (with a 10–15 s interval) every day for 4 d. A submerged (1.5 cm below the surface of the water, invisible to the animal) platform was fixed in the centre of a quadrant for all four trials each day, but the platform location was changed between days. Each mouse was allowed to stay on the platform for 30 s. Swimming paths for training session and probe test were monitored using an automatic tracking system. This system was used to record the swimming trace and calculate the latency to the platform.

#### Y-maze spontaneous alternation

The Y-maze apparatus was made of Plexiglas and had three identical arms (40×10×15 cm) placed at 120° with respect to each other. Specific motifs were placed on the walls of each arm, thus allowing visual discrimination. Each mouse was placed at the end of one arm and allowed to explore the apparatus freely for 5 min, with the experimenter out of the animal's sight. Total entries were scored as an index of ambulatory activity in the Y-maze. Alternations were defined as successive entries into each of the three arms on overlapping triplet sets. Percentage spontaneous alternation performance (SAP) was defined as the ratio of actual (total alternations) to possible (total arm entries - 2) alternations×100. The alternate arm returns (AARs) were also scored for each animal. If an animal went, for example, from arm A to arm B and back to arm A, one AAR was recorded.

### *In vivo* microdialysis

*In vivo* microdialysis measurements of extracellular dopamine and metabolites were performed in freely moving mice in an identical manner to that described before [23]. On the day of surgery, a mouse was anesthetized with chloral hydrate (400 mg/kg, i.p.) and placed in a stereotaxic apparatus. The concentric microdialysis probe (MD-2200, 2 mm membrane, Bioanalytical system, Inc., USA) was implanted into the right ventral striatum at the co-ordinates AP 0, L +2.2, V −2.0 mm according to the stereotaxic atlas. The extracellular levels of neurotransmitters in the mice striatum were measured at 7 d after surgery. For all groups, the probes were perfused with an artificial cerebrospinal fluid (ACSF, 140 mM NaCl, 2.7 mM KCl, 1.4 mM CaCl_2_, 1.2 mM MgCl_2_, 5.0 mM glucose, pH 7.4). Perfusion with ACSF started at 12 h after the probe insertion and lasted for 2 h at a rate of 2 µl/min for equilibrium. Afterward, dialysate samples were collected into polypropylene microcentrifuge vials at 20 min intervals. All samples were frozen at −70°C before they were analyzed. At the end of the experiments, the coronal sections of the brain were cut to verify the location of the probe. Dialysis samples were assayed for dopamine (DA), 3,4-dihydroxyphenylacetic acid (DOPAC), 4-hydroxy-3-methoxy-phenylacetic acid (HVA), and 5-hydroxyindoleacetic acid (5-HIAA) by high-performance liquid chromatography (HPLC) with electrochemical detection (ECD). The levels of neurotransmitters in the samples were calculated by extrapolating the peak area from a standard curve.

### *In vitro* Electrophysiology

Coronal slices (350 µm thick) containing prefrontal cortex (PFC) were prepared from 6–10 weeks old male WT or GAT1 KO littermate mice. After decapitation, the brain was removed and placed in oxygenated (95% O_2_/5% CO_2_) ACSF at 4°C. Slices were cut with a Leica VT1000S vibratome (Leica Instr. Ltd., Germany) and maintained at room temperature (23–25°C) in a holding chamber filled with oxygenated ACSF for at least 2 h, whereas slices for whole-cell recordings were initially incubated in warmed (32°C) ACSF for 30 min and then maintained at room temperature. Then a single slice was transferred to the recording chamber, where it was held between two nylon nets and continuously perfused with oxygenated ACSF (23–25°C) at a flow rate of 2–3 ml/min. The same ACSF was used in cutting, incubating and recording, and contained (in mM): 119 NaCl, 2.5 KCl, 2.5 CaCl_2_, 1 NaH_2_PO_4_, 1.3 MgSO_4_, 26.2 NaHCO_3_, and 11 D-glucose, saturated with 95% O_2_/5% CO_2_ (pH 7.4). The osmolarity of the ACSF was 310–320 mOsm/l. All electrophysiological recordings were performed at room temperature with an Axopatch-200B amplifier (Axon Instruments, CA) at the sampling rate of 10 kHz and filtered at 5 kHz. Data were acquired and analyzed using a Digidata 1322A interface and Clampfit 9.0 software (Axon Instruments, CA). Whole-cell recordings were made from the layer II/III pyramidal neurons of PFC. The neurons were visually identified using an upright microscope (BX51WI, Olympus, Japan) equipped with differential interference contrast optics and an infrared camera. Patch pipettes were made from borosilicate glass (1.5 mm OD) with a micropipette puller (PC-830, Narishige, Japan). The internal pipette solution for voltage-clamp recording contained (in mM): 140 KCl, 5 NaCl, 2 MgATP, 0.3 NaGTP, 0.1 EGTA, 10 HEPES. The pH was adjusted to 7.2, and the osmolarity was 300–310 mOsm/l. To block action potentials, 2 mM QX-314 was added into the pipette solution. The resistance of the patch electrode filled with above internal solution was 3–5 MΩ. Under voltage-clamp conditions, the cells were held at −70 mV. Series resistances were usually 10–20 MΩ. To record inhibitory postsynaptic currents (IPSCs), 10 µM CNQX and 20 µM d-APV were added to the ACSF to block glutamatergic responses. Tonic GABA_A_R-mediated currents were examined by applying the selective GABA_A_R antagonist picrotoxin into the slice chamber in a final concentration of 100 µM. The tonic GABA current was measured as the outward shift in the holding current.

### Western blot

The PFC from WT or GAT1 KO littermate mice was dissected according to the mouse brain atlas and were homogenized in the lysis buffer contained 20 mM Tris-Cl, pH 7.4, 150 mM NaCl, 1% Triton X-100, 1 mM EDTA, 3 mM NaF, 1 mM β-glycerophosphate, 1 mM Na_3_VO_4_ and 10% glycerol, with protease inhibitors (Sigma-Aldrich Co. LLC., USA). After lysed on ice for 30 min, the lysates were centrifuged at 13,000 g for 15 min at 4°C. The supernatants were collected and separated on 10% SDS-PAGE gels with 4% stacking gels. Then the proteins were transferred to polyvinylidene difluoride filters and incubated overnight with anit-GABA_A_R α1 (1∶200, Santa Cruz, USA), anit-GABA_A_R γ2 (1∶200, Santa Cruz, USA), anit-GABA_A_R α2 (1∶500, Chemicon, USA), anit-GABA_A_R α5 (1∶400, Chemicon, USA), anit-GABA_A_R β (1∶100, Chemicon, USA), anit-GABA_A_R δ (1∶200, Chemicon, USA) and anti-GAPDH (1∶4000, Kangchen, China). Secondary antibodies conjugated with horseradish peroxidase were then incubated with the filters at room temperature (23–25°C) for 2 h. After that the filters were visualized in ECL solution using the ImageQuant LAS 4000 mini Molecular Imaging System (GE Healthcare Life Sciences, USA) and their optic densities were analyzed via the Image J software (NIH, USA).

All drugs and chemicals in these experiments were purchased from Sigma (Sigma-Aldrich Co. LLC., USA). In the experiments with cortical slices, drugs were applied to the bathing medium. All the data were shown as the mean ± S.E.M., with statistical significance assessed by two-way AVONA and unpaired Student's *t*-test. All statistical analysis was performed using Origin 7.0 (OriginLab, USA).

## Results

### Locomotor hyperactivity and enhanced sensitivity to psychotomimetic drugs in GAT1 KO mice

**Figure 1:**
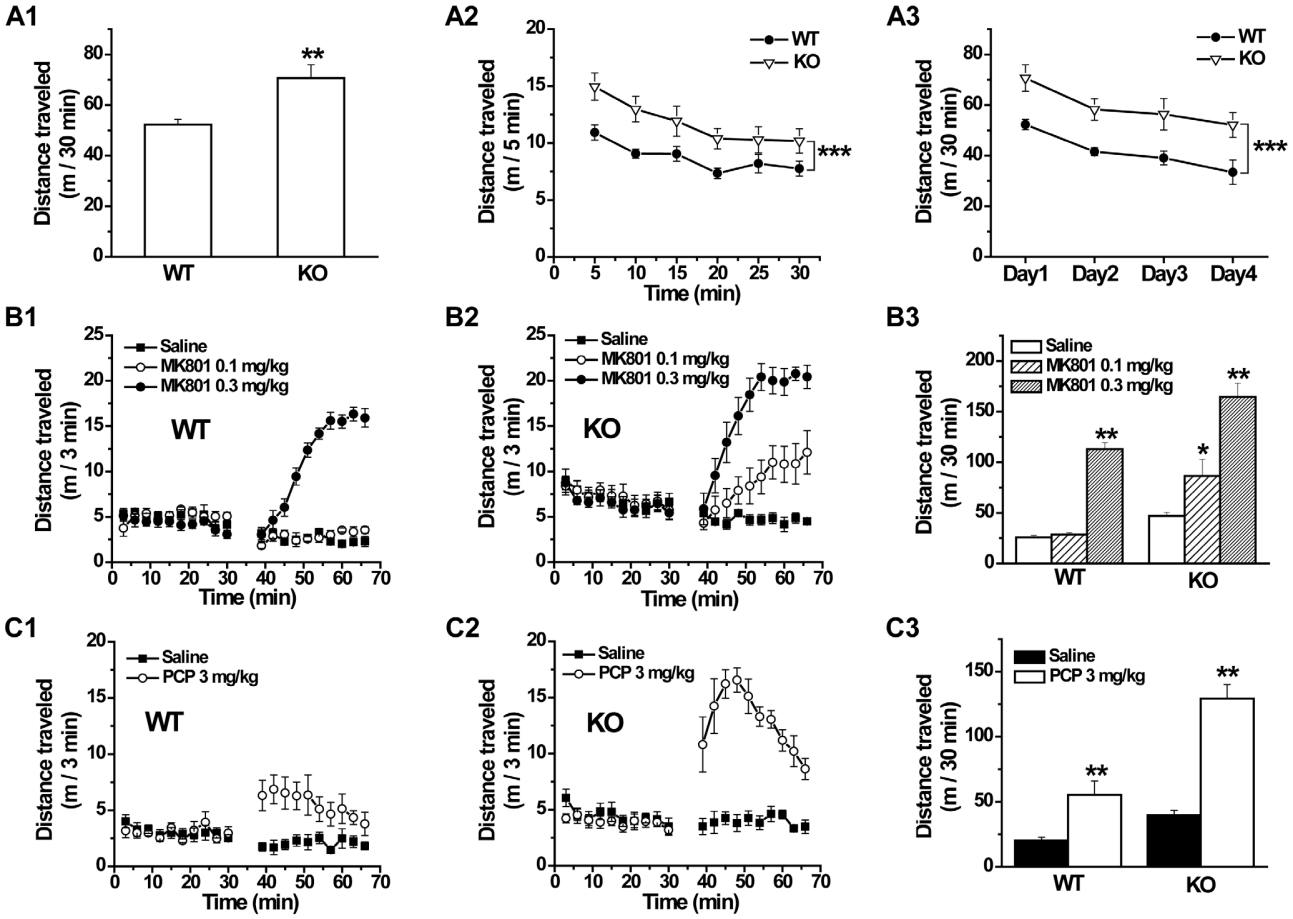
GAT1 knockout (KO) mice show locomotor hyperactivity and increased sensitivity to psychotomimetic drugs in the open field test. ***A1***, Total distance traveled of wild-type (WT) and knockout (KO) mice in 30-min tests. ***A2***, Distance traveled of WT and KO mice in each 5 min within the 30-min tests. ***A3***, Total distance traveled of WT and KO in 30-min tests repeated daily for 4 days. n = 8–9 for each genotype. ** *p*<0.01, *** *p*<0.001 vs WT, ANOVA. ***B*** and ***C***, To test the stimulant effects of NMDAR antagonist MK801 and phencyclidine (PCP), mice were injected i.p. with drug after 30-min acclimation in the open field, and then immediately tested for another 30 min. Locomotor hyperactivity could be induced by MK801 and PCP. GAT1 KO mice showed increased sensitivity to MK801 and PCP. ***B1***, ***B2***, ***C1*** and ***C2***, Distance traveled of WT and KO mice in each 3 min within 1 h tests. ***B3*** and ***C3***, Distance traveled of WT and KO mice in 30-min tests after drug administration. n = 6–8 for each group. * *p*<0.05, ** *p*<0.01 vs saline, ANOVA.

Schizophrenia is diagnosed on the basis of characteristic clinical syndromes in human individuals, including positive, negative and cognitive symptoms [24]. In rodents, various behavioral phenotypes have been proposed to be relevant to the symptoms of schizophrenia, such as the locomotor hyperactivity, abnormal social behaviors, sensorimotor gating deficits and cognitive impairment [25]. Therefore, to study whether GAT1 deficiency causes schizophrenia-related behavioral abnormalities, we first examined the locomotor activity of GAT1 KO mice in the open field test. We found that GAT1 KO mice showed significantly higher locomotor activity than WT in a single 30-min exposure to the open field (WT, 52.3±2.0 m; KO, 70.7±5.3 m; n = 8–9 for each genotype, *p*<0.01, *t*-test, Fig. 1A1), consistent with our previous finding [10]. Furthermore, all genotype mice showed similar short-term habituation within the 30-min test represented by the distance traveled per 5 min (genotype×time interaction, F(5, 84) = 0.498, *p*>0.1, ANOVA, Fig. 1A2) and similar long-term habituation within four consecutive daily tests (genotype×day interaction, F(3, 60) = 0.021, *p*>0.1, ANOVA, Fig. 1A3), while GAT1 KO mice showed persistent locomotor hyperactivity in both tests (short-term, F(1, 84) = 26.459, *p*<0.001, Fig. 1A2; long-term, F(1, 60) = 32.454, *p*<0.001, ANOVA, Fig. 1A3), suggesting that the locomotor hyperactivity in GAT1 KO mice is not due to the acute response to a novel environment.

Another feature of positive symptom in schizophrenia is the augmented response to psychotomimetic drugs, such as non-competitive NMDAR antagonists MK801 and PCP [26], [27]. Similarly, in rodents, locomotor hyperactivity can be induced by MK801 and PCP. As shown in Fig. 1B, MK801 at 0.3 mg/kg but not 0.1 mg/kg significantly increased the locomotor activity in WT mice (distance traveled in 30 min after drug treatment, saline, 25.9±1.9 m; MK801 0.1 mg/kg, 28.8±1.8 m; MK801 0.3 mg/kg, 113.0±6.4 m; n = 7–8 for each group, F(2, 19) = 137.9, 0.1 mg/kg vs saline, *p*>0.1; 0.3 mg/kg vs saline, *p*<0.01, ANOVA, Fig. 1B1 & 1B3), while in GAT1 KO mice, MK801 at 0.1 mg/kg was enough to induce locomotor hyperactivity (saline, 47.1±3.3 m; MK801 0.1 mg/kg, 86.5±16.4 m; MK801 0.3 mg/kg, 164.7±13.5 m; n = 7–8 for each group, F(2, 20) = 25.7, 0.1 mg/kg vs saline, *p* = 0.03; 0.3 mg/kg vs saline, *p*<0.01; genotype×dose interaction, F(2, 39) = 3.1, *p* = 0.05, ANOVA, Fig. 1B2 & 1B3). Another non-competitive NMDAR antagonist PCP (3 mg/kg) induced locomotor hyperactivity in both WT and GAT1 KO mice (WT, saline, 20.3±2.4 m, PCP, 55.3±10.7 m, F(1, 11) = 11.9, *p*<0.01; GAT1 KO, saline, 39.6±3.8 m, PCP, 129.3±10.8 m, F(1, 10) = 61.4, *p*<0.01, n = 6–7 for each group, ANOVA, Fig. 1C), while the stimulant effect was significantly larger in GAT1 KO mice (genotype×dose interaction, F(1, 21) = 12.9, *p*<0.01, ANOVA, Fig. 1C). These data indicate that GAT1 deficiency results in increased sensitivity to psychotomimetic drugs.

### Exaggerated responses to novel objects but impaired novel object recognition in GAT1 KO mice

**Figure 2:**
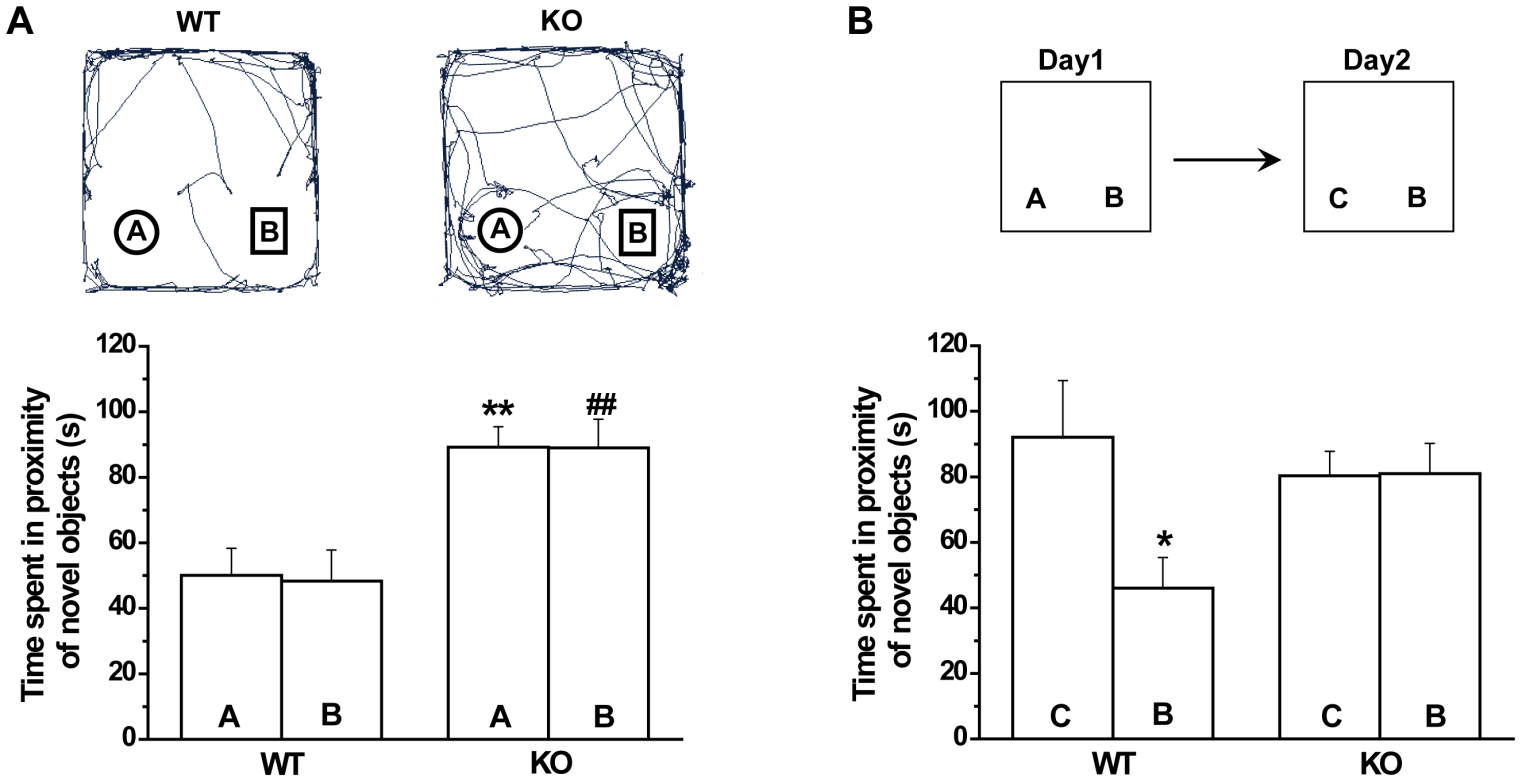
GAT1 KO mice show exaggerated locomotor responses to novelty but impaired object recognition memory. ***A***, GAT1 KO mice spent more time in the proximity of the novel objects A and B as compared to WT in 5-min tests. n = 12–13 for each group, ** *p*<0.01, ^##^ *p*<0.01 vs WT, ANOVA. ***B***, After 1 day, object A was changed to a novel object C. WT mice spent more time in the proximity of the object C than that of object B, indicating a object recognition memory. This memory was impaired in GAT1 KO mice. n = 12–13 for each group, * *p*<0.05 vs object C, ANOVA.

Then, we introduced two objects A and B into two distinct quadrants of the open field and counted the time spent near the objects by mice in a 5-min test. GAT1 KO mice spent significantly more time in the proximity of both objects than WT (object A, WT, 50.1±8.2 s, KO, 89.2±6.3 s, F(1, 23) = 14.6, *p*<0.01; object B, WT, 48.3±9.5 s, KO, 89.0±8.8 s, F(1, 23) = 10.0, *p*<0.01; n = 12–13 for each group, ANOVA, Fig. 2A). Both genotypes showed no preference for the two objects A and B (WT, F(1, 22) = 0.02, p>0.1; KO, F(1, 24) = 0.00, *p*>0.1, ANOVA; Fig. 2A). Then, the object recognition was tested 24 h later. One of the original objects (A) was replaced by a new object (C). WT mice showed a significant greater preference for the new object (object C, 92.1±17.3 s, object B, 46.0±9.3 s, n = 12, F(1, 22) = 5.5, *p* = 0.02, ANOVA, Fig. 2B), while GAT1 KO showed no such preference (object C, 80.3±7.4 s, object B, 81.0±9.2 s, n = 13, F(1, 24) = 0.00, *p*>0.1, ANOVA, Fig. 2B), indicating an impaired object recognition memory in the GAT1 KO mice.

### Abnormal social behaviors in GAT1 KO mice

**Figure 3:**
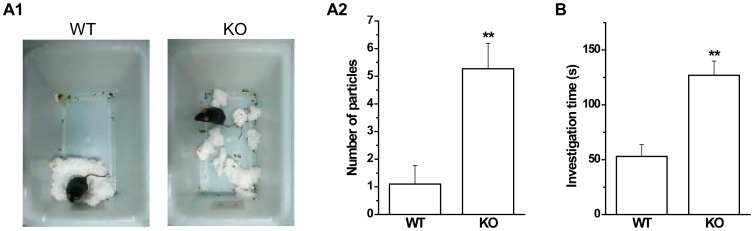
GAT1 KO mice show abnormal social behavior. ***A1***, Representative pictures of the cages of WT and KO mice. ***A2***, Statistical results showing the number of cotton particles in the cages. n = 9–11 for each genotype, ** *p*<0.01 vs WT, *t*-test. ***B***, GAT1 KO mice showed more social investigation time to an unfamiliar juvenile conspecific than that of WT mice. n = 9–10 for each genotype, ** *p*<0.01 vs WT, *t*-test.

Social behavioral tests in rodents are used to model negative symptoms of schizophrenia. Nesting is a common index of social behavior [28]. To examine the nesting behavior in GAT1 KO mice, we introduced ten cotton particles into an empty cage and then observed the formation of nest. We found that nests of GAT1 KO mice were poorly formed while WT mice usually formed a clean and identifiable nest (Fig. 3A1). The number of scattered cotton particles in the cages of GAT1 KO mice was significantly larger than that of WT mice (WT, 1.1±0.7; KO, 5.3±0.9; n = 9–11 for each genotype, *p*<0.01, *t*-test, Fig. 3A2). Then we examined another index of social behavior, the social interaction between the test mouse and a stranger juvenile conspecific. During a 10-min social interaction test, the total investigation time of GAT1 KO mice was significantly longer than that of WT mice (WT, 53.0±10.8 s; KO, 127.0±13.0 s; n = 9–10 for each genotype, *p*<0.01, *t*-test, Fig. 3B). These data suggest that GAT1 KO mice may have abnormal social behaviors, but also may possibly result from locomotor hyperactivity.

### Impaired prepulse inhibition and latent inhibition in GAT1 KO mice

**Figure 4:**
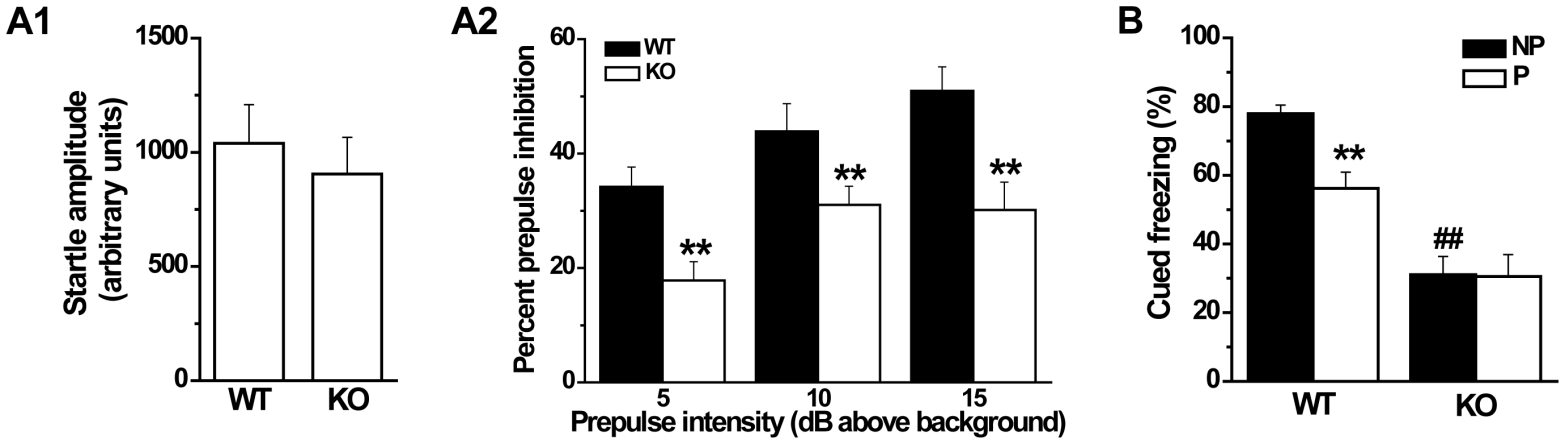
GAT1 KO mice show impaired prepulse inhibition and latent inhibition. ***A1***, Startle amplitude to a 120 dB acoustic stimulus was normal in GAT1 KO mice. ***A2***, GAT1 KO mice exhibited significantly reduced percent prepulse inhibition of the acoustic startle response across prepulse intensities (70, 75, 80 dB). n = 9–10 for each genotype, ** *p*<0.01 vs WT, ANOVA. ***B***, In cued fearing test, GAT1 KO mice showed reduced percent freezing. Pre-exposure to cue without shock significantly impaired the formation of fear memory in WT mice but not in GAT1 KO mice. n = 9–10 for each group, ^##^ *p*<0.01 vs WT, ** *p*<0.01vs NP, ANOVA.

Prepulse inhibition is a common measure of sensorimotor gating, which is decreased in schizophrenic patients [29]. PPI can also be tested in rodent models and represented by the attenuation of the startle response by a weak prepulse stimulus. We found that the basal startle amplitudes were not significantly different between genotypes at the startle stimulus only (120 dB) (WT, 1040±168; KO, 905±160; n = 9–10 for each genotype, *p*>0.1, *t*-test, Fig. 4A1). In all genotypes, PPI could be successfully detected in three different prepulse intensities (70, 75 and 80 dB), and increased with the elevated intensities (F(2, 72) = 4.363, n = 9–10 for each genotype, *p*<0.05, ANOVA, Fig. 4A2). However, the percent PPI was significantly lower in GAT1 KO mice than that of WT mice (F(1, 72) = 20.76, n = 9–10 for each genotype, *p*<0.01, ANOVA, Fig. 4A2), indicating the impaired sensorimotor gating in GAT1 KO mice.

Latent inhibition is another measure for the attentional function, and is abnormal in schizophrenic patients [30]. In rodents, LI can also be tested and refers to the ability to ignore biologically irrelevant stimuli. We used cued fear conditioning to examine LI in WT and GAT1 KO mice. In our previous study, we showed that the contextual fear memory was impaired in GAT1 KO mice [10]. Here we found that the cued fear memory was also significantly impaired in GAT1 KO mice (percent freezing, WT, 77.9±2.5%; KO, 31.1±5.3%; n = 9–10 for each genotype, F(1, 17) = 70.4, *p*<0.01, ANOVA, Fig. 4B), indicating an impairment of general cognitive functions in GAT1 KO mice. Pre-exposure to cue without shock significantly reduced the cued fear memory in WT mice (NP group, 77.9±2.5%; P group, 56.2±4.8%; n = 10 for each group, F(1, 18) = 18.2, *p*<0.01, ANOVA, Fig. 4B), but had no such effect in GAT1 KO mice (NP group, 31.1±5.3%; P group, 30.6±6.4%; n = 9–10 for each group, F(1, 17) = 0.00, *p*>0.1, ANOVA, Fig. 4B), suggesting the impaired attentional function in GAT1 KO mice.

### Impaired working memory in GAT1 KO mice

**Figure 5:**
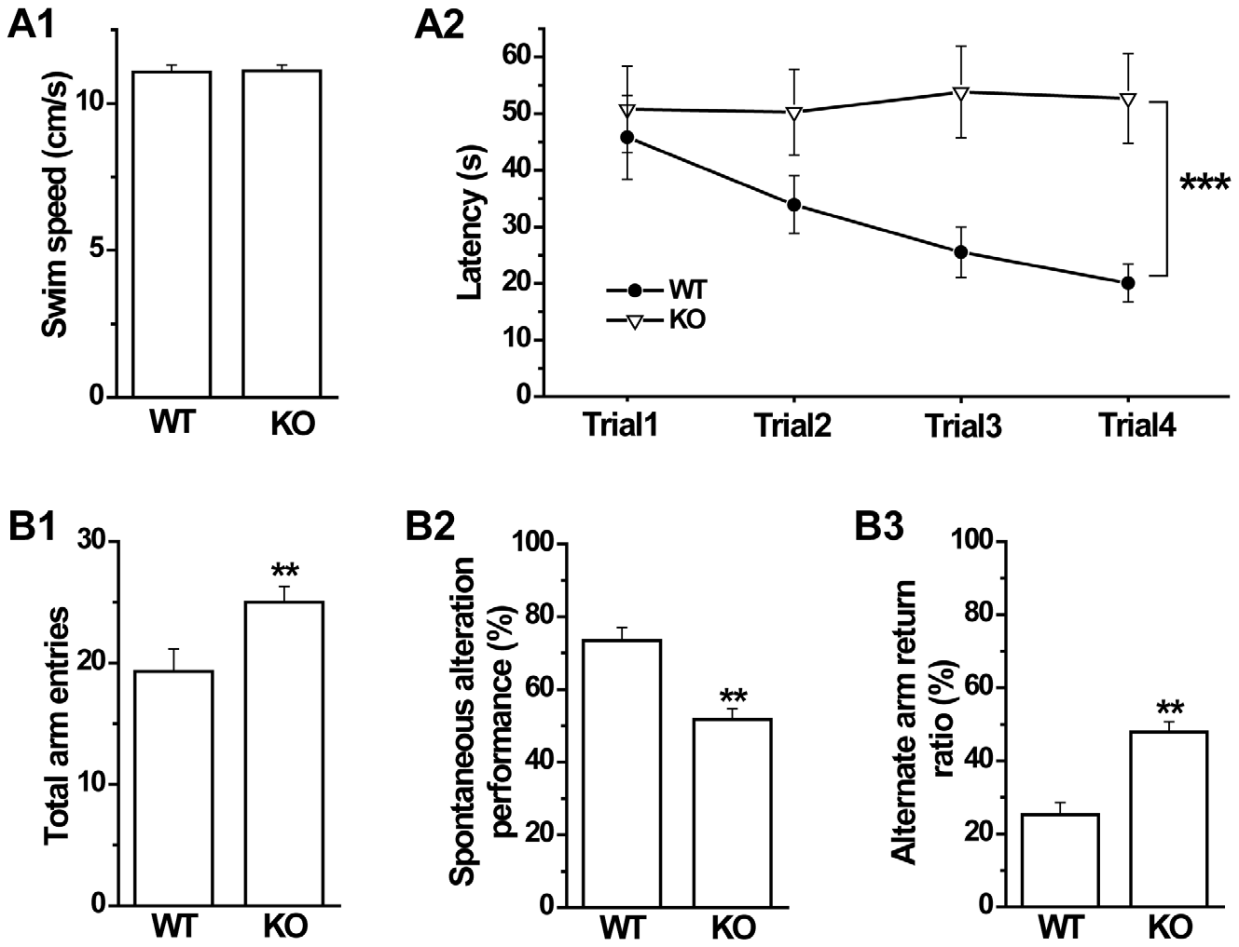
GAT1 KO mice show impaired working memory in Morris water maze and Y-maze tests. ***A1***, GAT1 KO and WT mice had similar swimming speeds. ***A2***, The latency to find the platform, plotted as function of trials, was significantly longer for GAT1 KO mice as compared to WT mice. n = 10–11 mice×4 days×4 trials for each genotype, ** *p*<0.001 vs WT, ANOVA. ***B1***, GAT1 KO mice showed significant more total arm entries than that of WT mice in Y-maze test. ***B2*** and ***B3***, The spontaneous alteration was significantly impaired in GAT1 KO mice, as shown by the reduced spontaneous alteration performance and increased alternate arm return ratio. n = 9–10 for each genotype, ** *p*<0.01 vs WT, *t*-test.

Cognitive impairment is an important feature in schizophrenia. In the previous study, we reported that GAT1 KO mice showed general long-term cognitive deficits in Morris water maze, passive avoidance and contextual fear conditioning tests [10]. In this study, we further examined the working memory function in GAT1 KO mice. First, we used Morris water maze to examine the short-term spatial working memory. Both genotypes had similar swimming speeds (WT, 11.07±0.23 cm/s; KO 11.10±0.20 cm/s; n = 10–11 mice×4 days×4 trials for each genotype, *p*>0.1, *t*-test, Fig. 5A1). However, WT mice but not GAT1 KO mice showed a significant decline of the latency in finding the platform within the four trials (genotype effect, F(1, 304) = 166.511, *p*<0.001; genotype×trial interaction, F(3, 304) = 15.329, *p*<0.001; n = 10–11 mice×4 days×4 trials for each genotype, ANOVA, Fig. 5A2), indicating the impaired spatial working memory of GAT1 KO mice.

Then, we examined the spontaneous alternation behavior in the Y-maze test, which is based on the animal's willingness to explore novel environmental stimuli and thought to reflect working memory performance. Consistent with the locomotor hyperactivity in open field, GAT1 KO mice exhibited more total arm entries in 5-min Y-maze tests than WT mice (WT, 19.3±1.9; KO 25.0±1.3; n = 9–10 for each genotype, *p*<0.01, *t*-test, Fig. 5B1). Meanwhile, GAT1 KO mice showed significant less spontaneous alternation but more alternate arm returns (SAP, WT, 73.4±3.6%; KO 51.8±2.9%; *p*<0.01, Fig. 5B2; AAR, WT, 25.3±3.3%; KO 47.8±2.9%; *p*<0.01, Fig. 5B3; n = 9–10 for each genotype, *t*-test), further indicating the impaired working memory of GAT1 KO mice.

### Reversal of locomotor hyperactivity by typical and atypical antipsychotic drugs

**Figure 6:**
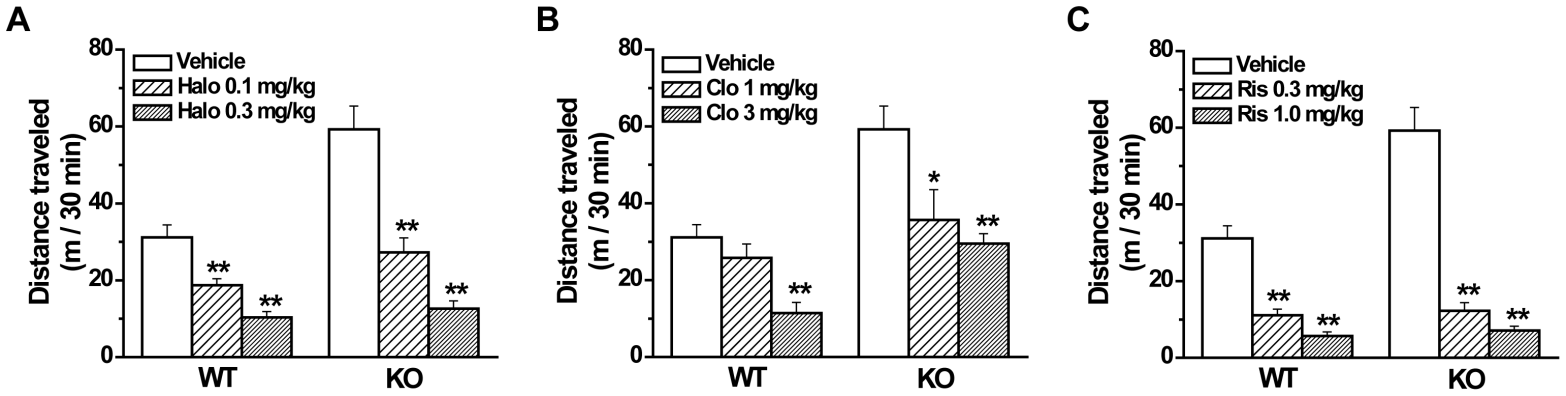
Typical (haloperidol) and atypical (clozapine and risperidone) antipsychotic drugs effectively reverse the locomotor hyperactivity in GAT1 KO mice. n = 7–9 for each group, * *p*<0.05, ** *p*<0.01 vs vehicle, ANOVA.

According to the above behavioral results, GAT1 KO mice displayed multiple behavioral abnormalities related to schizophrenic positive, negative and cognitive symptoms, suggesting that GAT1 KO mice may be a novel schizophrenia mouse model. Then we want to know whether commonly used antipsychotic drugs are effective in this mouse model. Here we chose a typical drug haloperidol and two atypical drugs clozapine and risperidone, and examined their effects on the locomotor hyperactivities of GAT1 KO mice. We found that in both WT and GAT1 KO mice, all three drugs could reduce the locomotor activity in a dose-dependent manner (haloperidol, F(2, 41) = 48.0, *p*<0.01; clozapine, F(2, 42) = 13.5, *p*<0.01; risperidone, F(2, 44) = 96.6, p<0.01; n = 7–9 for each group, ANOVA, Fig. 6). However, the decrease was greater in KO mice, especially for the haloperidol and risperidone treatment (genotype×dose interaction, haloperidol, F(2, 41) = 7.4, *p*<0.01; clozapine, F(2, 42) = 1.9, *p* = 0.09; risperidone, F(2, 44) = 13.2, *p*<0.01; n = 7–9 for each group, ANOVA, Fig. 6). These results indicate that GAT1 KO mice are more sensitive to antipsychotic drugs.

### Normal striatal dopamine levels in GAT1 KO mice

**Figure 7:**
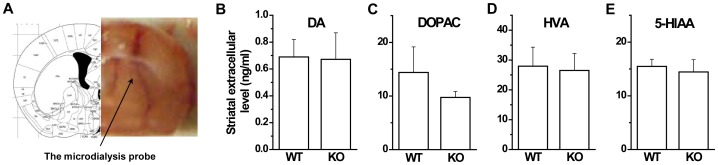
GAT1 KO mice show normal striatal dopamine levels. ***A***, The extracellular levels of dopamine and its metabolites were measured in the striatum of freely moving WT and GAT1 KO mice. ***B–E***, GAT1 mice had similar striatal levels of dopamine (DA) and its metabolites (DOPAC and HVA), as well as 5-HT metabolite (5-HIAA). n = 4–6 for each genotype, *t*-test.

Increased dopaminergic transmission has been considered to be a mechanism in schizophrenia. Furthermore, the antipsychotic drugs used in Fig. 6 have antagonism effects on dopaminergic receptors [31]. Thus, a possible mechanism is that GAT1 deficiency leads to increased dopaminergic tone. To test this hypothesis, we measured the extracellular levels of dopamine and its metabolites in the striatum of freely moving WT and GAT1 KO mice by combining in vivo microdialysis and HPLC (Fig. 7A). We did not find any significant differences in the levels of dopamine and its metabolites, as well as the 5-HT metabolite 5-HIAA between genotypes (DA, p>0.1; DOPAC, *p*>0.1; HVA, *p*>0.1; 5-HIAA, *p*>0.1; n = 4–6 for each genotype, *t*-test, Fig. 7B–7E), indicating no observable alterations in monoaminergic transmission. Therefore, locomotor hyperactivity and other aberrant behaviors in GAT1 null mice are not the result of alterations in monoaminergic transmission.

### Increased tonic GABA inhibition in the PFC of GAT1 KO mice

**Figure 8:**
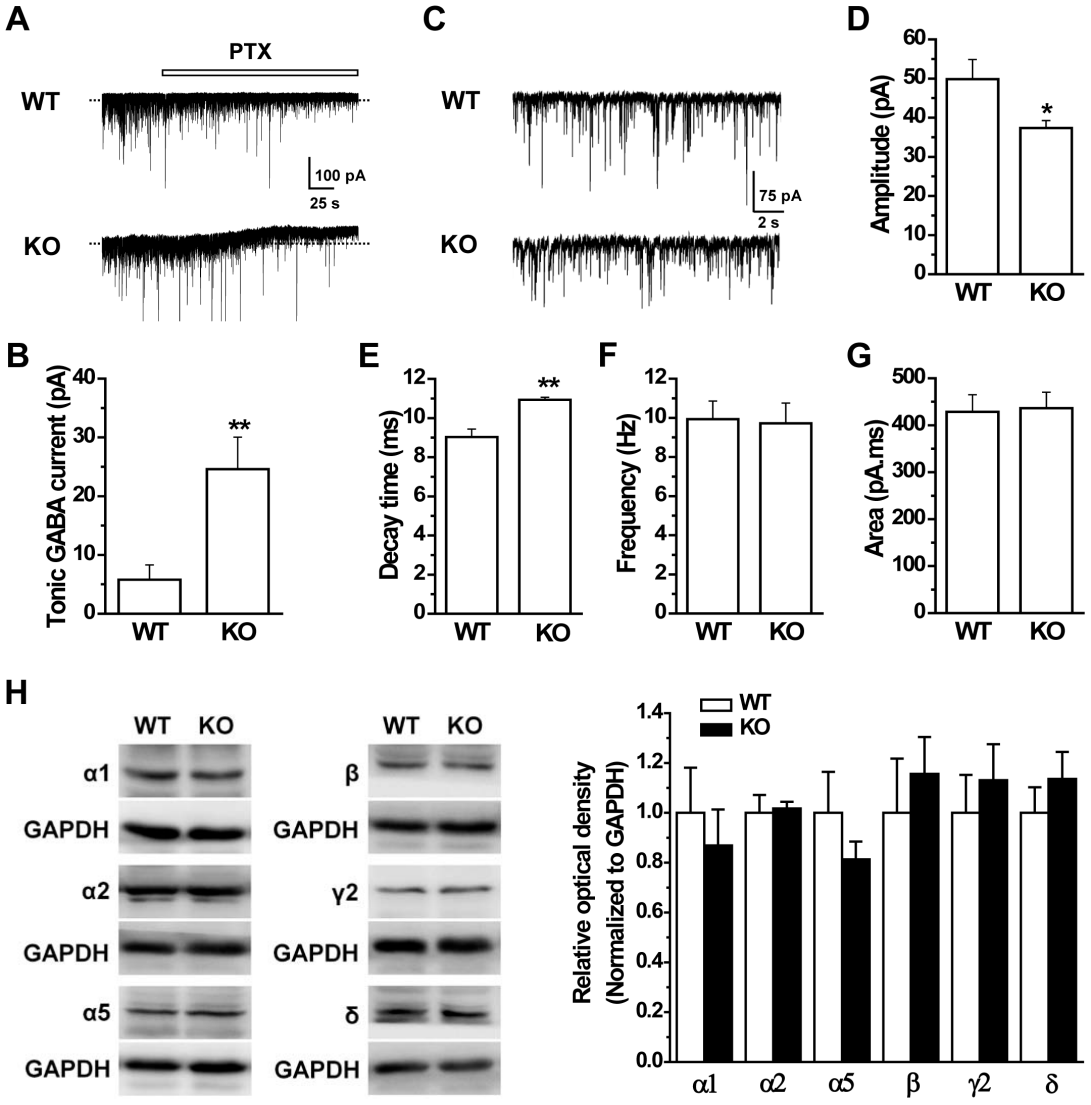
GAT1 KO mice show increased tonic GABA currents in PFC. ***A***, Representative traces showing the tonic GABA currents in pyramidal neurons of PFC. ***B***, Statistical results showing that the tonic GABA current was significantly larger in GAT1 KO mice. n = 5 for each group, ** *p*<0.01 vs WT, *t*-test. ***C***, Representative traces showing the sIPSCs in pyramidal neurons of PFC. ***D–G***, Statistical results of the amplitude (***D***), decay time (***E***), frequency (***F***) and current area (***G***) of sIPSCs. GAT1 KO mice showed decreased amplitude but increased decay time. The frequency and current area were not significantly changed. n = 7 for each group, * *p*<0.05, ** *p*<0.01 vs WT, *t*-test. ***H***, The expression of GABA_A_ receptor α1, α2, α5, β, γ2 and δ subunits remained unchanged in the PFC of GAT1 KO mice. *left*, representative immunoblots; *right*, statistic results. n = 4–5 for each group, *t*-test.

Since the dominant role of GAT1 in GABA reuptake and clearance, GAT1 deficiency can markedly change the efficacy of GABAergic inhibition. In the previous study, we have shown that GAT1 deficiency increased tonic GABA currents and prolonged GABAergic IPSCs in the hippocampus, which in turn modulated hippocampal synaptic plasticity and hippocampal-dependent learning and memory [10]. In this study, we examined the alterations of GABAergic transmission in PFC, the brain area which has been thought to be critical in schizophrenia. To distinguish the tonic GABA currents between WT and GAT1 KO slices, neither GAT1 inhibitors nor GABA were added. The GABA_A_ antagonist PTX (100 µM) failed to induce an obvious tonic current in WT slices (Fig. 8A and 8B), consistent with previous reports [32], [33], [34]. By contrast, 100 µM PTX treatment induced a significant tonic current in GAT1 null slices (WT, 5.8±2.4 pA; KO, 24.6±5.4 pA; n = 5 for each group, *p* = 0.01, *t*-test, Fig. 8A and 8B). We also analyzed the GABAergic spontaneous IPSCs (sIPSCs) and found that the amplitudes of sIPSCs were smaller in GAT1 KO mice than that of WT mice (WT, 49.8±5.0 pA; KO, 37.4±1.9 pA; n = 7 for each group, *p* = 0.03, *t*-test, Fig. 8D), but the decay time was significantly prolonged in KO mice (WT, 9.0±0.4 ms; KO, 10.9±0.1 ms; n = 7 for each group, *p*<0.01, *t*-test, Fig. 8E). Furthermore, there was no significant difference in frequency and current area between this two genotypes (frequency, WT, 9.9±0.9 Hz; KO, 9.7±1.0 Hz; n = 7 for each group, *p*>0.1, *t*-test, Fig. 8F; current area, WT, 428±36 pA ms; KO, 436±34 pA ms; n = 7 for each group, *p*>0.1, *t*-test, Fig. 8G). Taken together, these results indicate the increased tonic GABA inhibition in the PFC of GAT1 KO mice.

Decreased amplitude of sIPSC may result from the down-regulation of postsynaptic GABA_A_ receptors. To test this possibility, we examined the expression levels of the main GABA_A_ receptor subunits (α1, α2, α5, β, γ2 and δ) in the PFC of WT and GAT1 KO mice. All these subunits remained unchanged in the PFC of GAT1 KO mice compared to their WT littermates (n = 4–5 for each group, *p*>0.1, *t*-test, Fig. 8H), suggesting that reduced sIPSC amplitude was not due to downregulated postsynaptic GABA_A_ receptor.

### Reversal of locomotor hyperactivity and working memory defect by GABAergic antagonism

**Figure 9:**
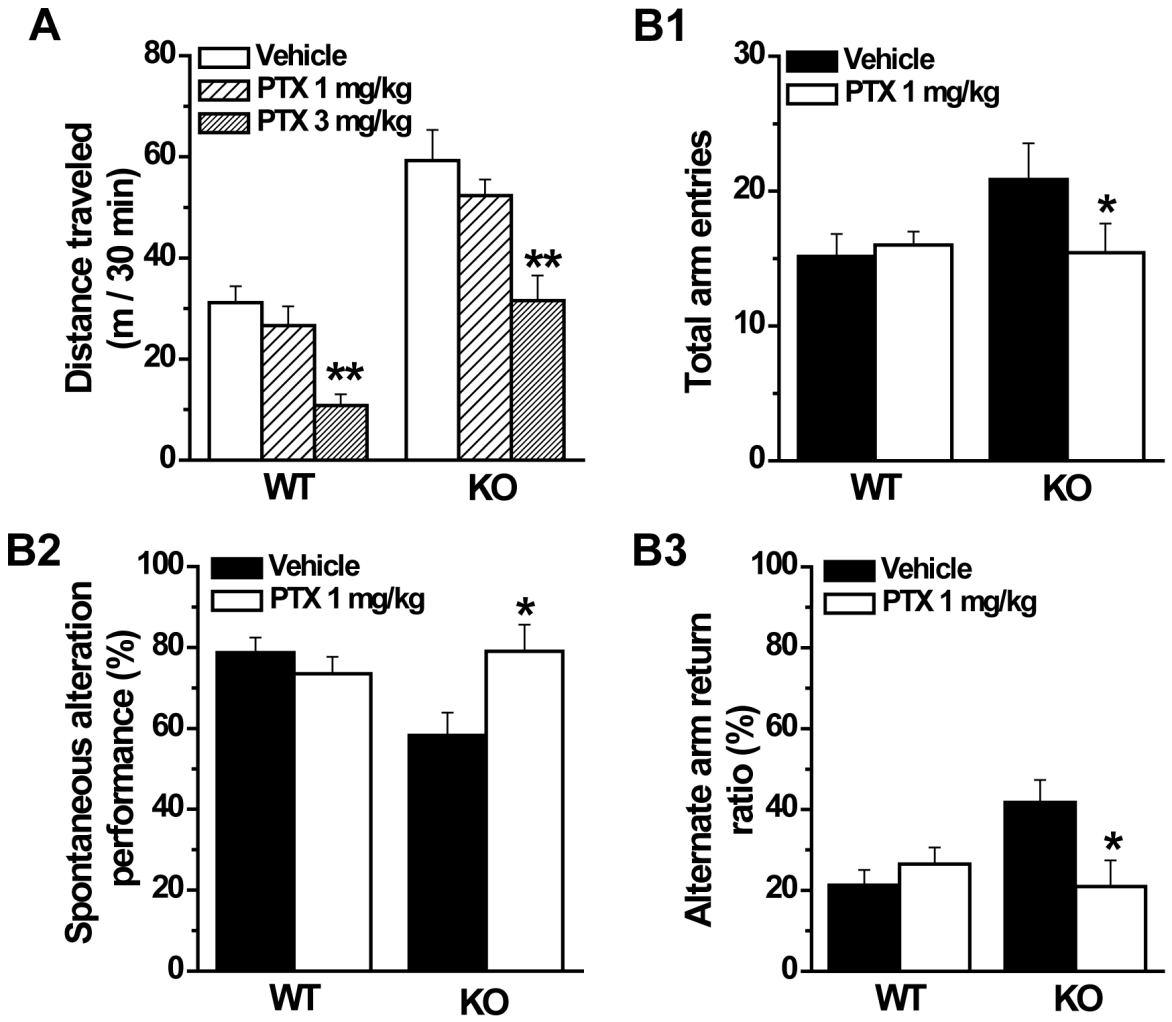
GABAergic antagonist picrotoxin ameliorates the locomotor hyperactivity and working memory defect of GAT1 KO mice. ***A***, PTX at 3 mg/kg reduced the locomotor hyperactivity in both WT and KO mice in open field. n = 7–8 for each group, ** *p*<0.01 vs vehicle, ANOVA. ***B1***, PTX at 1 mg/kg reduced the total arm entries of GAT1 KO mice in Y-maze test. ***B2*** and ***B3***, PTX at 1 mg/kg significantly increased the spontaneous alteration performance and reduced the alternate arm return ratio of GAT1 KO mice. n = 7 for each group, * *p*<0.05, vs vehicle, ANOVA.

The GABAergic inhibition is increased in the PFC and hippocampus, as well as cerebellum in the GAT1 KO mice. Thus, we want to know whether this increased GABAergic inhibition contributes to the schizophrenia-like behaviors. We used PTX to study the effect of GABAergic antagonism in GAT1 KO mice, and found that PTX at a dose below the threshold to induce seizure (3 mg/kg) could significantly alter locomotor activity in both WT and KO mice (F(2, 40) = 18.8, 3 mg/kg vs vehicle, *p*<0.01, ANOVA, n = 7–8 for each group, Fig. 9A). Then we studied the effect of PTX on the working memory using a dose of 1 mg/kg, at which PTX did not significantly reduce locomotor activity. We found that in Y-maze test, the same dose of PTX significantly reduced the total arm entries (vehicle, 20.9±2.7; PTX, 15.4±2.2; n = 7 for each group, F(1, 12) = 5.9, *p* = 0.03, ANOVA, Fig. 9B1), increased the spontaneous alternation (vehicle, 58.3±5.6%; PTX, 79.1±6.5%; F(1, 12) = 5.8, *p* = 0.03, ANOVA, Fig. 9B2) and reduced the AAR (vehicle, 41.7±5.6%; PTX, 20.9±6.5%; F(1, 12) = 5.8, *p* = 0.03, ANOVA, Fig. 9B3) in GAT1 null mice, but had no effect on the WT mice (total arm entries, F(1, 12) = 0.19, *p*>0.01; SAP, F(1, 12) = 0.86, *p*>0.1; AAR, F(1, 12) = 0.86, *p*>0.1; n = 7 for each group, ANOVA, Fig. 9B). These results indicate that GABAergic antagonism can treat the locomotor hyperactivity and working memory defect in GAT1 KO mice, suggesting that antagonizing GABAergic activity may be a potential strategy for the therapy of schizophrenia.

### Early onset of schizophrenia-like behaviors in GAT1 null mice

**Figure 10:**
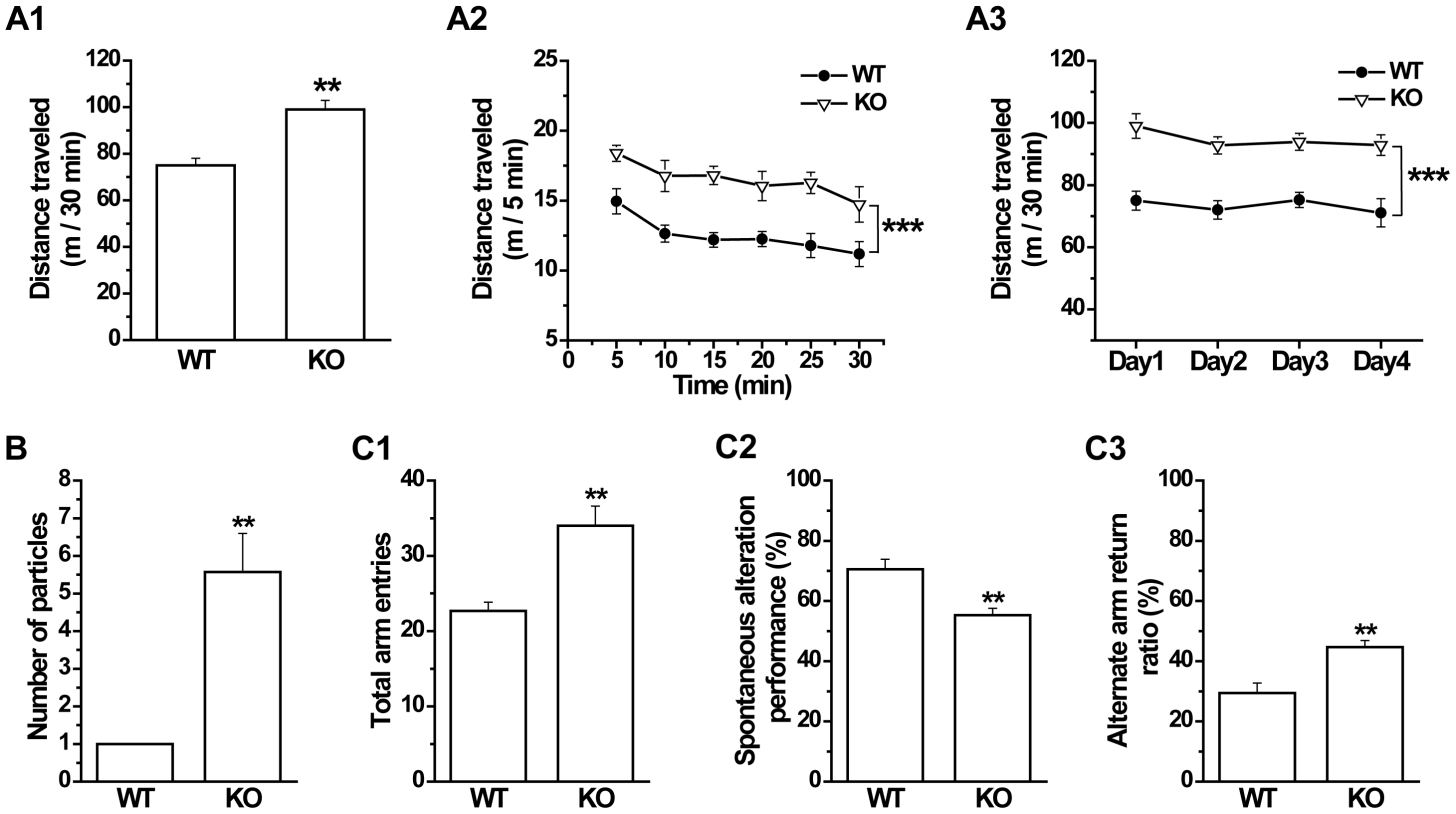
GAT1 KO mice show early onset of schizophrenia-like behaviors. ***A1***, Total distance traveled of young (4–5 weeks old) WT and KO mice in 30-min tests. ***A2***, Distance traveled of young WT and KO mice in each 5 min within the 30-min tests. ***A3***, Total distance traveled of young WT and KO mice in 30-min tests repeated daily for 4 days. n = 7–9 for each genotype. ** *p*<0.01, *** *p*<0.001 vs WT, ANOVA. ***B***, Young GAT1 KO mice showed increased number of cotton particles in the cages. n = 7–9 for each genotype. ** *p*<0.01 vs WT, *t*-test. ***C1***, Young GAT1 KO mice showed significant more total arm entries than that of WT mice in Y-maze test. ***C2*** and ***C3***, The spontaneous alteration was significantly impaired in young GAT1 KO mice, as shown by the reduced spontaneous alteration performance and increased alternate arm return ratio. n = 7–9 for each genotype, ** *p*<0.01 vs WT, *t*-test.

Schizophrenia is a neurodevelopmental disorder which is closely associated with environmental and developmental vulnerability factors [24]. Unlike other genetic neurodevelopmental disorders that occur early after birth, schizophrenia is especially rare in children under the ages of 7–8 years old. While about 4% of schizophrenia cases occur before eighteen years old (early-onset schizophrenia, EOS), most individuals manifest psychotic symptoms in late adolescence or early adulthood (adult-onset schizophrenia, AOS) [35]. To examine the emergent time of the schizophrenia-like symptoms in GAT1 KO mice, we performed open field, nesting behavior and Y-maze spontaneous alternation tests on young (4–5 weeks old) mice, which mimics the early adolescence in human [36]. We found that young GAT1 KO mice showed significantly higher locomotor activity than WT ones while exposing to the open field (WT, 75.0±3.0; KO, 99.0±3.9; *p*<0.01, *t*-test, Fig. 10A1; F(1, 84) = 69.37, *p*<0.001, ANOVA, Fig. 10A2; F(1, 56) = 81.067, *p*<0.001, ANOVA, Fig. 10A3; n = 7–9 for each genotype). When testing mice's nesting behavior, we found that the number of scattered cotton particles in the cages of GAT1 KO mice was significantly larger than that of WT mice (WT, 1.0±0.0; KO, 5.6±1.0; n = 7–9 for each genotype, *p*<0.01, *t*-test, Fig. 10B), suggesting abnormal social behaviors. Furthermore, young GAT1 KO mice exhibited more total arm entries (WT, 22.7±1.2; KO, 34±2.6; n = 7–9 for each genotype, *p*<0.01, *t*-test, Fig. 10C1), less spontaneous alternation (WT, 70.6±3.3; KO, 55.3±2.2; n = 7–9 for each genotype, *p*<0.01, *t*-test, Fig. 10C2) and more alternate arm returns (WT, 29.4±3.3; KO, 44.7±2.2; n = 7–9 for each genotype, *p*<0.01, *t*-test, Fig. 10C3) in 5-min Y-maze tests than WT mice, indicating the impaired working memory. Taken together, these data suggested that GAT1 KO mice exhibited schizophrenia-related behavioral abnormalities early in the adolescence. Since EOS represents a chronic and possibly more severe variant of AOS [35], [37], the early onset of schizophrenia phenotype of GAT1 null mice suggested the critical role of GAT1 in the pathogenesis of schizophrenia.

## Discussion

### The schizophrenia-like behavioral phenotypes of GAT1 KO mice

In previous studies, GAT1 KO mice have been found to have several behavioral abnormalities, including altered responses to ethanol [22], reduced anxiety and depression [38], reduced aggressive behaviors [39], and impaired long-term memory [10]. In this study, using more comprehensive behavioral tests, we found that the same mice exhibited locomotor hyperactivity and increased sensitivity to psychotomimetic drugs, abnormal social behaviors, impaired attentional function as measured by PPI and LI, as well as defects of working memory. These behavioral phenotypes were reminiscent of schizophrenic positive, negative and cognitive symptoms. Firstly, the persistent locomotor hyperactivity and increased responses to novel objects in GAT1 KO mice mimic the positive symptom “psychomotor agitation” in schizophrenic patients [25]. Psychotomimetic drugs, such as non-competitive NMDAR antagonists MK801 and PCP can exacerbate the psychomotor agitation in schizophrenia [26], [27], such as their effects in GAT1 KO mice. Secondly, social withdrawal is a prominent negative symptom in schizophrenia. As a social activity, nest-building was dramatically impaired in GAT1 KO mice. Defects in social behavior were also reported previously, as reflected by reduced aggression in GAT1 KO mice [39]. Thirdly, the cognitive symptom of schizophrenia was mimicked by the impaired working memory and previously characterized long-term memory defects in GAT1 KO mice [10]. Finally, GAT1 KO mice showed impaired PPI and LI, which relate to the deficits of sensorimotor gating and attentional function in schizophrenia.

### GABAergic mechanism in schizophrenia

Besides dopaminergic and glutamatergic hypotheses, GABAergic mechanism has attracted increasing attentions in the study of schizophrenic pathogenesis. Although genetic studies have yielded little in the study of GABAergic system in schizophrenia, postmortem human studies have found out GABAergic disturbances in the brain of schizophrenic patients. These changes include reduced mRNA level and expression of GAD67 and GAT1, and upregulation of GABA_A_R α2 subunit in the PFC in schizophrenic patients [3]. Especially, GAD67 and GAT1 were downregulated in almost identical cellular patterns, only in a subset of parvalbumin-expressing interneurons, called chandelier cells, which project axo-axonic synapse to the axon initial segments of pyramidal neurons [3], [11], [40]. In the postsynaptic site, GABA_A_R α2 subunit is predominantly expressed in the pyramidal neuron axon initial segments, and upregulated in schizophrenic patients [41]. Thus, in this axo-axonic synapse, the changes could reflect deficient inhibition due to reduced presynaptic GABA synthesis or excessive inhibition due to increased postsynaptic GABA_A_Rs and decreased GABA reuptake. It has been supposed in several studies that GAD67 downregulation is the dominant mechanism in schizophrenia while the increase of GABA_A_R and the decrease of GAT1 are likely to be compensatory alterations [3], [21]. The idea was supported by a clinical trial that the GABA_A_R α2 agonist could improve prefrontal cognitive function in schizophrenic patients [42]. However, the change of GABA level in schizophrenia is not conclusive. A recent study showed elevated GABA levels in the anterior cingulated cortex of schizophrenic patients [19], although normal and reduced GABA levels have also been reported [14], [15], [16], [17], [18]. Moreover, Neuregulin 1, a susceptibility gene of schizophrenia, has been shown to increase the release of GABA [43]. In addition, in the axo-axonic synapse, GABA may not exert the classical inhibitory role, but may have an excitatory action due to the high intracellular chloride concentrations in axons [44]. Thus, the role of GABA_A_R α2 agonist may be more complicated in terms of producing inhibition. Furthermore, several atypical antipsychotics, such as clozapine and olanzapine, can antagonize GABA_A_R in the cortex and decrease extracellular GABA in the PFC [45].

In most neurological diseases, such as epilepsy, ischemia, chronic pain, also including schizophrenia, GABAergic disinhibition has been thought as a main pathogenic mechanism. However, several recent studies showed that tonic GABA inhibition was enhanced in epilepsy and ischemia, while reducing the increased tonic GABA inhibition was effective in the treatment of both diseases [46], [47]. These findings strongly suggest that the function of tonic GABA inhibition in disease conditions is different, even opposite to that of phasic (synaptic) GABAergic inhibition. Our study may also support this hypothesis in schizophrenia. We found that GAT1 KO mice showed significant higher tonic GABA currents in the PFC area than that of WT mice, while GABAergic antagonism could treat the locomotor hyperactivity and working memory defect in GAT1 KO mice. Thus, our study provides more evidence of the novel function of tonic GABA inhibition in neurological diseases. Thus, future explorations of the exact role of GABA and GAT1 in schizophrenia would require more detailed observation and analysis using brain region- and cell type-specific GAT1 conditional KO mice.

Neural oscillations play a fundamental role in coordinated activity during normal brain functioning [48]. Thus they're the crucial targets in schizophrenia research [49], [50], [51]. Neural oscillations contribute critically to establish precise temporal correlations between distributed neuronal responses [48], [49]. Clinical studies also reported that both gamma and theta oscillation were impaired in schizophrenic patients [49], [50], [51]. GABA-mediated transmission was reported to produce synchronized network oscillations which are hypothesized to be essential for normal cognitive function [49], [50], [51]. Compared to gamma oscillation, theta oscillation was much less explored in neural disorders so far [48], [49]. We previous found hippocampal theta oscillation was impaired in GAT1 null mice [10]. Thus it may sever as a potential explanation for those cognitive symptoms. Additionally, schizophrenia is also considered as a neurodevelopment disorder [52], [53]. A recent study in mouse model showed that the synergistic interaction between depolarizing GABA signaling and DISC1 appeared to affect risk for schizophrenia [54], indicating that elevated extracellular GABA may also contribute to the pathogenesis of schizophrenia. Given that ambient GABA was increased in GAT1 KO mice, a similar mechanism may also exist in this animal model.

### Relation to glutamatergic and dopaminergic mechanisms

As the major inhibitory system in the brain, GABAergic system has important functions in modulating glutamatergic and dopaminergic transmissions, which are largely involved in the pathogenesis of schizophrenia. For instance, hypofunction of NMDAR has been proposed as an underlying cause in schizophrenia pathophysiology [55]. Blockade of NMDAR can elicit psychotic symptoms in humans that are similar to those seen in schizophrenic patients [26]. In a rodent model, selective knockout of NMDAR in corticolimbic interneurons leads to schizophrenia-like behaviors [56]. In the previous study, we found that GAT1 activity modulated NMDAR-dependent neural plasticity in the hippocampus [10]. Thus, the enhanced tonic GABA inhibition in the PFC by GAT1 KO may also suppress the activation of NMDAR by modulating the local depolarization. Indeed, we found that GAT1 KO mice showed increased sensitivity to non-competitive NMDAR antagonists MK801 and PCP, suggesting an impaired NMDAR function in GAT1 KO mice. Although the expression levels of major NMDAR subunits (GluN1, GluN2A and GluN2B) remained unchanged [10], we cannot exclude the possibility that the membrane trafficking or the phosphorylation level of these receptors change due to the chronic elevation of GABAergic inhibition in GAT1 null mice. Moreover, non-NMDARs such as α-amino-3-hydroxy-5-methyl-4-isoxazolepropionic acid receptors (AMPARs) and metabotropic glutamate receptors (mGluRs) also play critical roles in the pathogenesis of schizophrenia. For example, AMPAR subunit GluA2 was reduced in the brains of patients with schizophrenia [57], [58] Increased group I mGluR subunit mGluR5 [59] and reduced group II mGluR subunit mGluR3 expression [60] were reported in this illness. Furthermore, LY379286, an mGluR 2/3 agonist, can block the PCP induced hyperlocomotion [61] and reverse the MK-801-induced NMDAR dysfunction [62]. Apparently, additional experiments are required to examine the possible involvement of NMDARs, AMPARs or mGluRs in the development of schizophrenia-like behaviors in GAT1 null mice.

The GABAergic efficacy can also modulate dopamine activity, but in a more complicated manner, depending on the intensity of GABA manipulation and pre-existing dopamine activity [45], [63], [64]. In this study, we did not find any change in the levels of dopamine and its metabolites, although increased striatal dopamine has been detected in schizophrenic patients in several studies [65], [66], [67]. In fact, NMDAR knockdown mice and calcineurin KO mice also showed several schizophrenia-like behaviors, but yet have normal striatal dopamine levels [68], [69]. Here, we cannot exclude the possibility that the dopamine release under some stimuli is changed in GAT1 KO mice. Furthermore, we found that several antipsychotic drugs with dopamine receptor antagonism effects could reverse the locomotor hyperactivity in GAT1 KO. Whether the dopamine receptor activity is altered by GAT1 KO remains to be investigated.

### Rodent models for schizophrenia

Animal models are useful tools in studying the pathogenesis and treatment in human diseases. Although several symptoms of schizophrenia are difficult to measure directly in rodents, various behavioral tests in rodents have been established with potential relevance to the symptoms of schizophrenia [25]. Indeed, the schizophrenia-like behaviors have been observed and characterized in some rodent models. First, dopaminergic manipulation such as direct injection of dopamine receptor agonists [70] and dopamine transporter knockout or knockdown [71], [72] can model certain aspects of schizophrenia. Second, schizophrenia-related behaviors have been found in mice with mutations in components of glutamatergic system, such as NMDAR [56], [69], NMDAR glycine binding site [73], glycine transporter [74], metabotropic glutamate receptor [75], [76] and calcineurin [68]. Third, there are several mouse models of schizophrenia based on the mutations of susceptibility genes, such as Neuregulin 1 and Erb4 [77], [78], [79], as well as Disrupted-In-Schizophrenia 1 [80], [81], [82], [83], [84]. In addition, rats with neonatal ventral hippocampal lesions also develop a rodent model for schizophrenia [85]. In this study, GAT1 KO mice showed behaviors relevant to almost all symptoms of schizophrenia, and were sensitive to the commonly used antipsychotic drugs, indicating a novel mouse model for schizophrenia, which may provide new insights in studying schizophrenia etiology and developing new antipsychotic drugs.
